# Predictors of adherence to electronic self-monitoring in patients with bipolar disorder: a contactless study using Growth Mixture Models

**DOI:** 10.1192/j.eurpsy.2023.1072

**Published:** 2023-07-19

**Authors:** A. Ortiz, C. Park, C. Gonzalez-Torres, M. Alda, D. Blumberger, I. Husain, M. Sanches, B. H. Mulsant

**Affiliations:** 1 Department of Psychiatry, University of Toronto; 2 CAMH, Toronto; 3 Dalhousie University, Halifax, Canada

## Abstract

**Introduction:**

Several studies have reported on the feasibility and impact of e-monitoring using computers, or smartphones, in patients with mental disorders, including Bipolar Disorder (BD). Despite some promising early results, concerns have been raised about the motivation and ability of patients with BD to adhere to e-monitoring, in particular when they are depressed or manic. While studies on e-monitoring have examined the role of demographic factors, such as age, gender, or socioeconomic status and use of health apps, to our knowledge, no study has examined clinical characteristics that might impact adherence with e-monitoring of patients with BD.

**Objectives:**

We analyzed adherence to e-monitoring in patients with BD who participated in an ongoing e-monitoring study and evaluated whether demographic and clinical factors would predict adherence.

**Methods:**

Eighty-seven participants with BD in different phases of the illness were included. Patterns of adherence for wearable use, daily and weekly self-rating scales over 15 months were analyzed to identify adherence trajectories using growth mixture models (GMM). Multinomial logistic regression models and Multiple Component Analyses were fitted to compute the effects of predictors on GMM classes.

**Results:**

Adherence rates were 79.5% for the wearable; 78.5% for weekly self-ratings; and 74.6% for daily self-ratings. GMM identified three latent class subgroups: (i) participants with good adherence with the protocol; (ii) participants with partial adherence; (iii) participants with poor adherence. Women, participants with a history of suicide attempt, and those with a history of inpatient admission were more likely to belong to the group with good adherence.

**Conclusions:**

Participants with higher illness burden (e.g., history of admission to hospital, history of suicide attempts) have higher adherence rates to e-monitoring. This is important because our findings debunk myths around illness burden as an obstacle to adhere to e-monitoring studies. Participants might have seen e-monitoring as a tool for better documenting symptom change and better managing their illness, thus motivating their engagement.

**Disclosure of Interest:**

None Declared

